# Isolation and identification of *Salmonella* spp. from red foxes (*Vulpes vulpes*) and badgers (*Meles meles*) in northern Italy

**DOI:** 10.1186/s13028-014-0086-7

**Published:** 2014-12-10

**Authors:** Mario Chiari, Nicola Ferrari, Daniele Giardiello, Paolo Lanfranchi, Mariagrazia Zanoni, Antonio Lavazza, Loris G Alborali

**Affiliations:** Istituto Zooprofilattico Sperimentale della Lombardia e dell’Emilia Romagna “Bruno Ubertini”, Via Bianchi 7/9, 25124 Brescia, Italy; Department of Veterinary Sciences and Public Health, Università degli Studi di Milano, via Celoria 10, 20133 Milan, Italy

**Keywords:** *Salmonella* spp, Opportunistic carnivores, Red fox, Badger

## Abstract

**Background:**

*Salmonella* spp. have been isolated from a wide range of wild animals. Opportunistic wild carnivores such as red foxes (*Vulpes vulpes*) and badgers (*Meles meles*) may act as environmental indicators or as potential sources of salmonellosis in humans. The present study characterizes *Salmonella* spp. isolated from the intestinal contents of hunted or dead red foxes (n = 509) and badgers (n = 17) in northern Italy.

**Findings:**

Thirty-one strains of *Salmonella* belonging to 3 *Salmonella enterica* subspecies were isolated. Fourteen different serovars of *S. enterica* subsp. *enterica* were identified, among which were serovars often associated with human illness.

**Conclusions:**

Wild opportunistic predators can influence the probability of infection of both domestic animals and humans through active shedding of the pathogen to the environment. The epidemiological role of wild carnivores in the spread of salmonellosis needs to be further studied.

## Findings

*Salmonellae* are enteric bacteria capable of infecting humans and both domestic and wild animals [1]. The Center for Disease Control and Prevention and the European Food Safety Authority reports that salmonellosis is the most important foodborne infection in humans in industrialized countries [2]. Although *Salmonella* infections in humans are generally transmitted through food of animal origin, such as eggs, chicken, pork or beef meat [3], the role of wildlife in the maintenance of salmonellosis is of increasing interest. *Salmonella* spp. can be shed in faeces from healthy animals for a long period of time and can be isolated at virtually every step of the game meat chain [4]. In addition, wildlife can be involved in human salmonellosis taking part in the ecology of these bacteria and thereby contributing to the persistence of bacteria in the environment [5].

In the last two decades, the populations of badgers (*Meles meles*) and, in particular, red foxes (*Vulpes vulpes*) have increased and the animals have adapted to peri-urban and urban environments across Europe. Ecological factors have been attributed to the increase in fox population, in particular the abundant availability of anthropogenic food sources [6,7]. Red foxes and badgers can be considered as indicators and spreaders of zoonotic infections due to their feeding habits [8]. Indeed, they have an omnivorous diet that includes prey and plants and they also scavenge around human waste disposal sites and dustbins. Thus, they are exposed to many potential sources of *Salmonella* [9], considering also the fact that *Salmonella* spp. can survive for long periods in the soil, water, and on a variety of surfaces [10,11]. Presence of *Salmonella* spp. in the intestinal contents of red foxes and badgers may reflect true intestinal infections and potential excretion of bacteria thus suggesting the role of these species in the ecology of *Salmonella* spp. [12].

Plenty of literature on the prevalence of *Salmonella* spp. in humans, livestock and more recently, wild ungulates [13-15] is available, while on the contrary little is known and scarce data are available about the natural occurrence and spread of this pathogen in small carnivores.

In order to investigate the prevalence of *Salmonella* infections in the red fox and badger populations in northern Italy, a total of 509 hunted or found dead (including road kills) red foxes and 17 found dead (including road kills) badgers were examined. The sampling was carried out within a health monitoring program on free-ranging animals in the Lombardy Region in northern Italy from 2009 to 2010 and included both rural and peri-urban habitats. Out of the 509 foxes examined, age was reported in114, while the gender was recorded in 182 (98 adults and 16 un-weaned cubs; 100 males and 82 females).

No lesions related to salmonellosis as expected were found at necropsy neither in foxes nor in badgers as salmonellosis is often subclinical [12].

Contents of the large intestine were sampled and cultured according to the International Organization for Standardization (ISO) 6579:2002/Amd 1:2007 method (ISO 2007) for *Salmonella* spp. Isolation [16]: Intestinal contents (25 g) were transferred to sterile sampling bags with 225 ml of buffered peptone water and incubated at 37°C for 24 h (pre-enrichment phase). Thereafter, 0.1 ml was inoculated on a Modified Semisolid Rappaport Vassiliadis (MSRV; Oxoid, Hampshire, UK) media and incubated for 48 h at 41.5°C. *Salmonella* spp. suspected colonies were then plated on two selective solid media: Xylose Lysine Deoxycholate agar (XLD; bioMérieux, Bagno a Ripoli, Italy) and Brilliant Green Agar (BGA; Vacutest Kima, Arzergrande, Italy). All presumptive *Salmonella* spp. isolates were confirmed using biochemical tests (BBL™ Enterotube™ II, Becton Dickinson, Heidelberg, Germany). Complete serological characterization of the *Salmonell*a strains was performed. This included rapid slide agglutination test for the detection of somatic antigens (Statens Serum Institut, Copenhagen, Denmark) and the hot tube agglutination test (Becton Dickinson, Heidelberg, Germany) for the determination of flagellar antigen. The results of the antigen determination were used for the final serological characterization using the White -Kauffmann - Le Minos scheme.

The prevalence of enteric *Salmonella* infection was calculated for each species. The exact Fisher’s test was performed to evaluate the association between sex, age and the isolation of bacteria in foxes. All confidence intervals (CI) were implemented using the Clopper-Pearson method setting 1-α = 0.95 as the confidence level.

**Figure 1:**
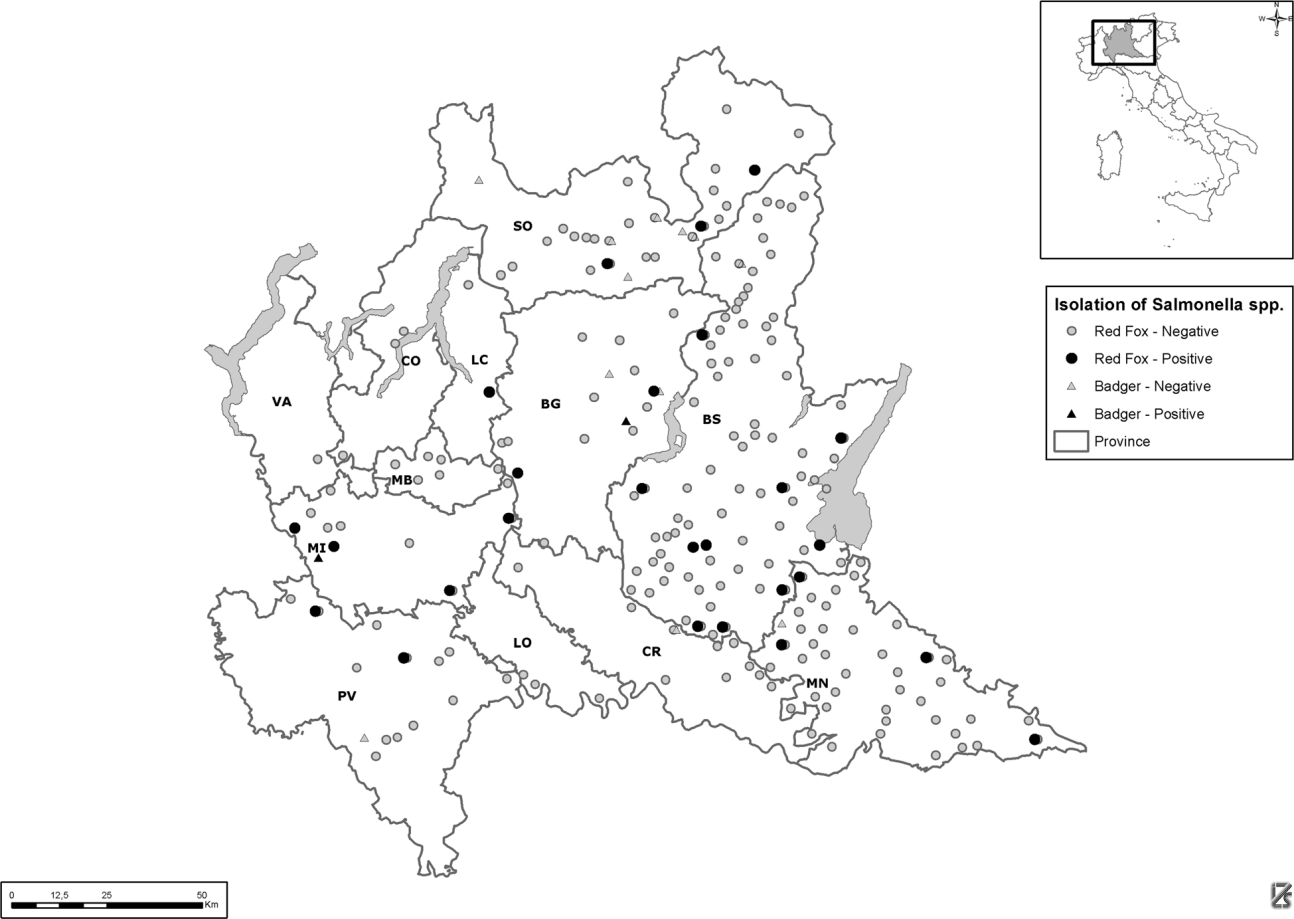
**Spatial distribution of positive and negative** ***Salmonella*** **spp. isolation from red foxes (** ***Vulpes vulpes*** **) and badgers (** ***Meles meles*** **) in Lombardy region, Italy.** A total of 509 hunted or found dead red foxes and 17 found dead badgers were examined for *Salmonella* spp. by culturing as part of a health monitoring program for free-ranging animals established in the Lombardy region of Italy from 2009 to 2010, including both rural and peri-urban habitats.

*Salmonella* spp. were isolated from 29 foxes (5.70%; 95% CI: 3.85% - 8.08%) and 2 badgers (11.76%; CI 95% 1.46% - 36.44%) coming from the Italian Lombardy region (Figure 1).

A prevalence of 8.00% was found in male foxes (95% CI: 3.52% to 15.16%) while no females tested positive (0.00%; 95% CI: 0.00 - 4.40%) (Table 1). Sex was significantly associated with isolation of *Salmonella* spp. (*P* = 0.01). Eight adult foxes were positive (8.16%; 95% CI: 3.59% - 15.45%) while no un-weaned cubs were found positive (0.00%; 95% CI: 0.00 to 20.59%) (Table 1). No statistical association between age class and isolation of *Salmonella* spp. was found (*P* = 0.60).

**Table 1:** **Number and prevalence of** ***Salmonella*** **positive foxes (** ***Vulpes vulpes*** **) in the Lombardy region, Italy**

		**No of samples**	**Positive**	**Prevalence**
	Female	82	0	0,00% (0.00% - 4.40%)
Sex	Male	100	8	8.00% (3.51% - 15.16%)
	Not recorded	327	21	6.42% (4.02% - 9.65%)
	Adult	98	8	8.16% (3.59% - 15.45%)
Age group	Young	16	0	0.00% (0.00% - 20.59%)
	Not recorded	395	21	5.32% (3.32% - 8.01%)

Isolated *Salmonella* strains included 16 different serotypes in red foxes and 2 in badgers, belonging to three different subspecies of *Salmonella enterica* strains, i.e. *S. enterica* subsp. *enterica* (27 strains, 14 serovars), *S. enterica* subsp. *diarizonae* (1 strain, 1 serovars) and *S. enterica* subsp. *houtenae* (1 strain, 2 serovars) (Table 2). In particular, *S*. Typhimurium, which is often involved in cases of human salmonellosis [17], was identified in nine animals.

**Table 2:** ***Salmonella*** **spp. isolated from red foxes (** ***Vulpes vulpes*** **) and badgers (** ***Meles meles*** **) in the Lombardy Region, Italy**

***S. enterica*** **serovars**	**Red fox**	**Badger**	**Total**
*S. enterica* subsp*. enterica*	25	2	27 (87.09%)
*S.* Typhimurium	8	1	9 (29.03%)
*S.* Infantis	0	1	1 (3.22%)
*S.* Derby	1	0	1 (3.22%)
*S.* Enteritidis	2	0	2 (6.45%)
*S.* Mbandaka	1	0	1 (3.22%)
*S.* Enterica 4,12:i	3	0	3 (9.67%)
*S.* Napoli	1	0	1 (3.22%)
*S.* Ohio	1	0	1 (3.22%)
*S.* Anatum	1	0	1 (3.22%)
*S.* Livingston	1	0	1 (3.22%)
*S.* Hessarek	1	0	1 (3.22%)
*S.* Muenchen	1	0	1 (3.22%)
*S.* Thompson	3	0	3 (9.67%)
*S.* Veneziana	1	0	1 (3.22%)
*S. enterica* subsp. *diarizonae* (1 serovars)	1	0	1 (3.22%)
*S. enterica* subsp. *houtenae* (2 serovars)	3	0	3 (9.67%)
Total	29	2	31

The prevalence of enteric salmonellosis found in foxes in the present study corresponds to observations in Norway where 6.5% of 215 red foxes were *Salmonella* positive [12], but only four serotypes were identified in the Norwegian study (*S*. Typhimurium 4,12:i:1,2, *S*. Hessarek, *S*. Kottbus and *S*. IIIb:61:k:1,5,(7)).

The observed prevalence of *Salmonella* infections in red foxes and badgers were unexpectedly low considering their omnivore feeding behaviour and the high prevalence found in wild boars living in the same areas (24.82%) [15]. *Salmonella* spp. have also been found in prevalences ranging from 5.5% to 24.82% in wild boars in other areas of Europe [1,4,15,18]. The difference in prevalence observed between foxes and wild boars may be explained by the different social behavior of these species. Indeed, the effect of animal social structure at the local scale is recognized to influence the transmission of infections between individuals [19]. Since *Salmonella* spp. are transmitted via a fecal-oral route, the maintenance of the infection in wild boars may be favored by large social groups that amplified the chance of contact between different individuals [20]. On the contrary foxes are characterized by small familiar groups defending their territories thus making contact between individuals less intimate.

Infection of red foxes with *S*. Typhimurium has been described as the result of ingestion of *Salmonella* infected, sick or dead small passerines during winter [12]. Moreover, carriage of *S*. Enteritidis by foxes near poultry farms has been related to their predatory and scavenging habits [21].

Five serovars of *Salmonella* subsp. *enterica* (i.e. *S*. Enteritidis, *S*. Typhimurium, *S*. Infantis, *S*. Derby, *S*. Mbandaka) represented 45.1% of the isolates. It is noteworthy that these serovars are among the 10 serovars responsible for most human cases of salmonellosis in Europe during 2010 [2]. This finding may reflect that foxes have extended their habitats into urban and peri-urban areas and therefore may be exposed to the same serovars as humans [6].

In conclusion, *Salmonella* spp. were isolated from the large intestine of 5.70% of the red foxes and 11.76% of the badgers in the Italian Lombardy region. Serovars representing 45.1% of the isolates were among the 10 serovars responsible for most cases of human salmonellosis in Europe in 2010. Consequently, wild predators, through an active shedding of the pathogen in the environment, can indirectly increase the probability of infection of both domestic animals and humans.
